# Supplementary material for: Observation of quantum many-body effects due to zero point fluctuations in superconducting circuits
Source: Nat Commun. 2019 Nov 20;10:5259. doi: 10.1038/s41467-019-13199-x (PMC6868017; doi:10.1038/s41467-019-13199-x)
Supplement: Supplementary file 1 — Supplementary Information [file 41467_2019_13199_MOESM1_ESM.pdf]

Supplementary Note 1: Experimental setup

The measurement setup is displayed in Supplementary Figure 1. The samples are put in a dilution refrigerator with a 25mK base temperature. $|S_{21}|$ is measured using a Vector Network Analyzer (VNA). An additional microwave source was used for two-tone measurements, while a global magnetic field was applied via an external superconducting coil. Both the coil and the sample were held inside a mu-metal magnetic shield coated on the inside with a light absorber made out of epoxy loaded with silicon and carbon powder. IR filters are 0.40mm thick stainless steel coaxial cables. The bandwidth of the measurement setup goes from 2.5 GHz to 12 GHz.

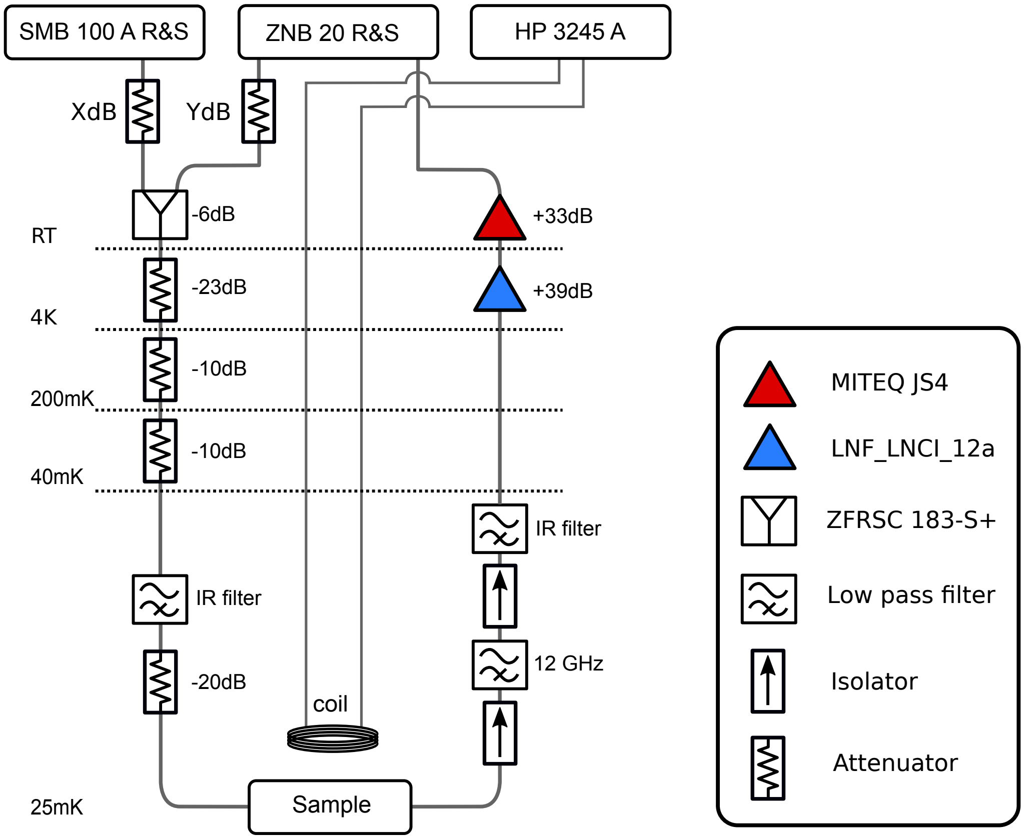

Supplementary Figure 1. **Measurement setup.**

Supplementary Note 2: Odd and Even modes

Our device consists of two long Josephson chains of $N+1$ sites tailored in the linear regime (with Josephson energy $(\hbar/2e)^2/L$ much larger than the capacitive energy) interconnected via a smaller Josephson junction or weak-link (operating in the regime of small Josephson energy $E_{J,\text{bare}}$). Linearizing the tunneling term within each chain, but keeping the non-linear coupling between them, the Hamiltonian of the system reads:

$$\hat{H} = \frac{(2e)^2}{2}\sum_{i,j=0}^{N}\sum_{\sigma,\sigma'\in L,R}\hat{n}_{i\sigma}[C]^{-1}_{i,\sigma,j,\sigma'}\hat{n}_{j,\sigma'} + \frac{1}{2}\frac{\hbar^2}{(2e)^2 L}\sum_{i=1}^{N-1}\sum_{\sigma\in L,R}\left(\hat{\phi}_{i,\sigma}-\hat{\phi}_{i+1,\sigma}\right)^2 - E_{J,\text{bare}}\cos\left(\hat{\phi}_{0,L}-\hat{\phi}_{0,R}\right), \quad (1)$$

with $\hat{n}_{i,\sigma}$ and $\hat{\phi}_{i,\sigma}$ the charge and phase operators on site $i \in [1..N]$ and in chain $\sigma = L, R$. These operators are canonically conjugate and obey at the quantum mechanical level the commutation rules $\left[\hat{\phi}_{i,\sigma}, \hat{n}_{j,\sigma'}\right] = i\delta_{i,j}\delta_{\sigma,\sigma'}$. The capacitance matrices can be read off the equivalent circuit in Supplementary Figure 2, and are decomposed into an intra-chain part $[C_0] = [C]_{LL} = [C]_{RR}$ and an interchain part intra-chain part $[C_1] = [C]_{LR} = [C]_{RL}$, which read explicitely:

$$
[C_0] = \begin{bmatrix}
C_\mathrm{I} & -C & 0 & 0 & 0 & \dots & 0 \\
-C & 2C + C_\mathrm{g} & -C & 0 & 0 & \dots & 0 \\
0 & -C & 2C + C_\mathrm{g} & -C & 0 & \dots & 0 \\
\vdots & \vdots & \ddots & \ddots & \ddots & \dots & 0 \\
0 & 0 & 0 & -C & 2C + C_\mathrm{g} & -C & 0 \\
0 & 0 & 0 & 0 & -C & 2C + C_\mathrm{g} & -C \\
0 & 0 & 0 & 0 & 0 & -C & C_\mathrm{O}
\end{bmatrix},
$$

and $[C_1]_{i,j} = -\delta_{0,i}\delta_{j,0}(C_\mathrm{J} + C_\mathrm{sh})$ with $(i,j) \in [0,N]^2$. The total capacitance at the weak-link end of the chain amounts to $C_\mathrm{I} = C_\mathrm{J} + C_\mathrm{sh} + C + C_\mathrm{g}$, while the capacitance at the connecting output port is $C_\mathrm{O} = C_\mathrm{c} + C_\mathrm{c,I} + C$.

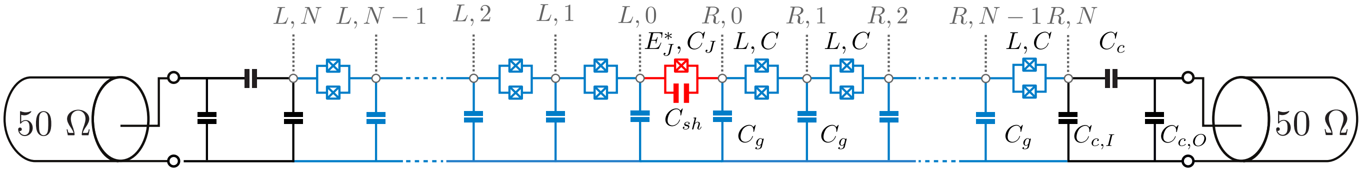

Supplementary Figure 2. **Electrical circuit of the device.** The capacitance network is indicated for the output ports (in black), the two chains (in blue) and the weak link (in red).

Due to the symmetry of our device, it is useful to define respectively even and odd modes:

$$
\hat{n}_{j,\pm} = \frac{1}{2}\left(\hat{n}_{j,R} \pm \hat{n}_{j,L}\right), \tag{2}
$$

$$
\hat{\phi}_{j,\pm} = \left(\hat{\phi_{j,R}} \pm \hat{\phi_{j,L}}\right). \tag{3}
$$

In this basis, the Hamiltonian decomposes in two uncoupled subsystems: $\hat{H} = \hat{H}_+ + \hat{H}_-$, where:

$$
\hat{H}_+ = \frac{(2e)^2}{2} \sum_{i,j=0}^{N} \hat{n}_{i,+} \left[\frac{C_0 + C_1}{2}\right]^{-1}_{i,j} \hat{n}_{j,+} + \frac{1}{4}\frac{\hbar^2}{(2e)^2 L} \sum_{i=1}^{N-1} \left(\hat{\phi}_{i,+} - \hat{\phi}_{i+1,+}\right)^2, \tag{4}
$$

$$
\hat{H}_- = \frac{(2e)^2}{2} \sum_{i,j=0}^{N} \hat{n}_{i,-} \left[\frac{C_0 - C_1}{2}\right]^{-1}_{i,j} \hat{n}_{j,-} + \frac{1}{4}\frac{\hbar^2}{(2e)^2 L} \sum_{i=1}^{N-1} \left(\hat{\phi}_{i,-} - \hat{\phi}_{i+1,-}\right)^2 + E_\mathrm{J}\left(1 - \cos\hat{\phi}_{0,-}\right). \tag{5}
$$

$\hat{H}_+$ reduces to the Hamiltonian of a linear chain, while $\hat{H}_-$ takes the form of a boundary Sine-Gordon-like model.

Supplementary Note 3: Fitting the transmission resonances

The transmission spectrum consists of pairs of peaks, that are fitted according to the model described in the Methods section of the main text. Close to a pair of even/odd resonances, the transmission is given by the formula:

$$
S_{21} = \frac{i\kappa_\mathrm{ext}(\omega_\mathrm{o} - \omega_\mathrm{e})}{\left(\kappa_\mathrm{ext} + \kappa_\mathrm{o} + -2i(\omega - \omega_\mathrm{o})\right)\left(\kappa_\mathrm{ext} + \kappa_\mathrm{e} - 2i(\omega - \omega_\mathrm{e})\right)}, \tag{6}
$$

with ω_o and ω_e the even/odd resonance frequencies, κ_e and κ_o their respective intrinsic damping rate, and κ_ext the broadening due to the 50 Ω output ports. A large selection of fitted spectra (for all three samples and various temperatures) is shown in Supplementary Figure 3.

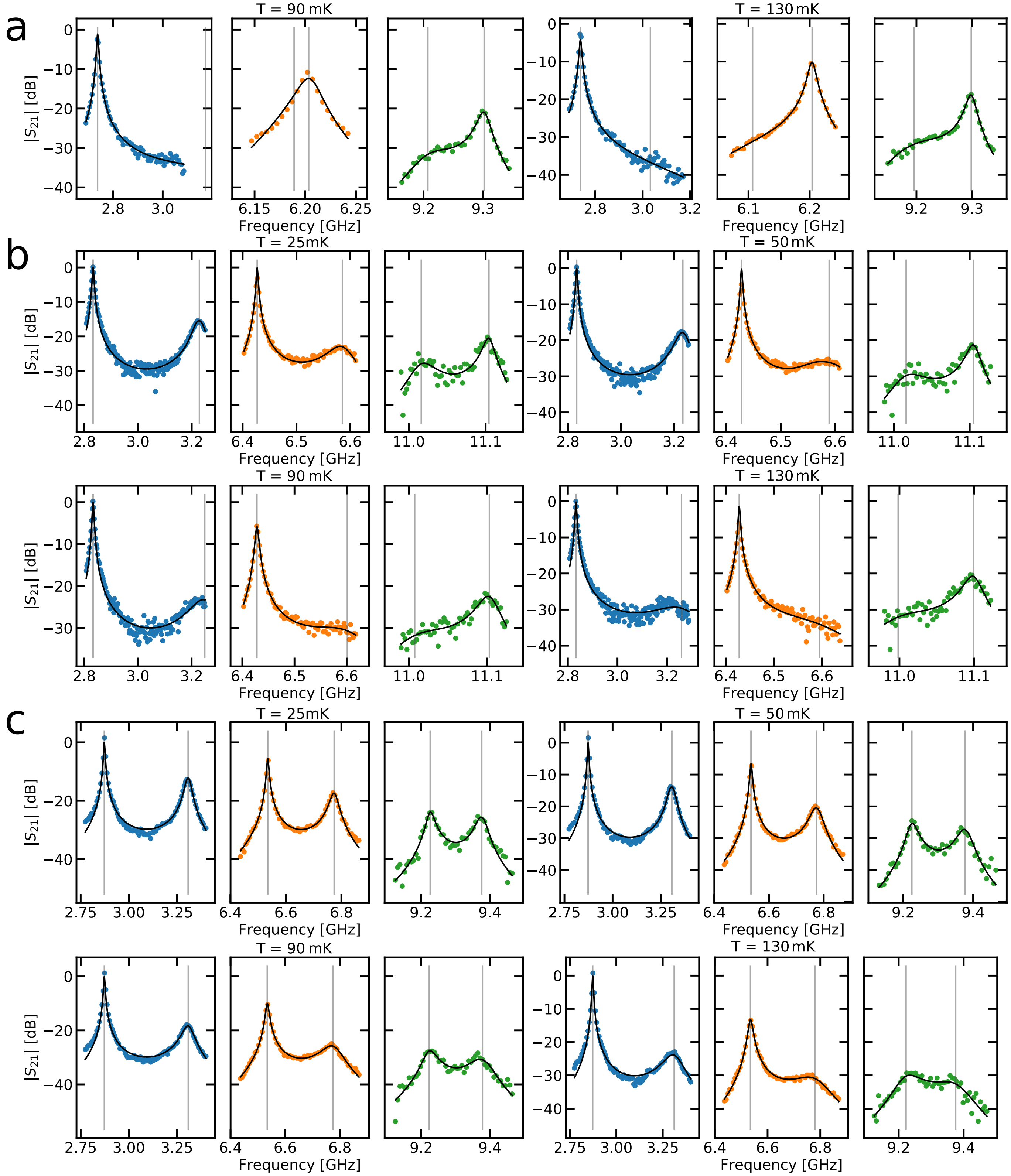

Supplementary Figure 3. **Fitting of various peak pairs**. Panel **a** is for sample 300, panel **b** is for sample 375 and panel **c** is for sample 450. Various temperature choices are indicated, and for each case, three frequencies ranges are indicated (in blue, orange and green respectively). Vertical grey lines are the positions of the resonance pairs found by the regression (black lines) using formula (6).

Supplementary Note 4: The self consistent harmonic approximation

The hamiltonian $\hat{H}_-$ describes a quantum many-body problem that cannot be solved analytically, and we therefore develop here an approximate yet microscopic approach to the problem. From now on, we will discard the - index in all fields, and replace $\hat{\phi}_{0,-}$ by $\hat{\phi}_{\rm J}$. The self consistent harmonic approximation (SCHA) is used to find the approximate ground state at thermal equilibrium [1, 2]. This method consist of finding the best harmonic Hamiltonian $\hat{H}_{\rm t}$ which satisfies the Gibbs-Bogoliubov inequality $F \leq F_{\rm t} + \langle \hat{H} - \hat{H}_{\rm t} \rangle_{\rm t}$, where:

$$F_{\rm t} = -k_{\rm B} T \ln Z_{\rm t}, \tag{7}$$

$$Z_{\rm t} = {\rm tr}\Big(e^{-\hat{H}_{\rm t}/k_{\rm B}T}\Big), \tag{8}$$

$$\langle \hat{H} - \hat{H}_{\rm t} \rangle_{\rm t} = {\rm tr}\Big((\hat{H} - \hat{H}_{\rm t})\hat{\rho}_{\rm t}\Big), \tag{9}$$

$$\hat{\rho}_{\rm t} = \frac{1}{Z_{\rm t}} e^{-\hat{H}_{\rm t}/k_{\rm B}T}. \tag{10}$$

The trial Hamiltonian $\hat{H}_{\rm t}$ is defined by replacing in $\hat{H}$ the non-linear tunneling term $-E_{\rm J}\cos\hat{\phi}_{\rm J}$ by a renormalized potential $E_{\rm J}^*\hat{\phi}_{\rm J}^2/2$. The physical reason is that the zero point fluctuations of the small junction explore a large part of the Josephson potential, which amounts in first approximation to lower its effective Josephson energy from the bare value $E_{\rm J}$ to a renormalized value $E_{\rm J}^*$. Explicitely, the trial Hamiltonian reads:

$$\hat{H}_{\rm t} = \frac{(2e)^2}{2} \sum_{i,j=0}^{N} \hat{n}_i [C]^{-1}_{i,j} \hat{n}_j + \frac{1}{2}\frac{\hbar^2}{(2e)^2} \sum_{i,j=0}^{N} \hat{\phi}_i [L^{-1}]_{i,j} \hat{\phi}_j, \tag{11}$$

with the capacitance matrix:

$$[C] = \frac{1}{2}\begin{bmatrix} C_\Sigma & -C & 0 & 0 & 0 & \dots & 0 \\ -C & 2C + C_{\rm g} & -C & 0 & 0 & \dots & 0 \\ 0 & -C & 2C + C_{\rm g} & -C & 0 & \dots & 0 \\ \vdots & \vdots & \ddots & \ddots & \ddots & \dots & 0 \\ 0 & 0 & 0 & -C & 2C + C_{\rm g} & -C & 0 \\ 0 & 0 & 0 & 0 & -C & 2C + C_{\rm g} & -C \\ 0 & 0 & 0 & 0 & 0 & -C & C_{\rm O} \end{bmatrix},$$

where $C_\Sigma = C_{\rm I} + C_{\rm J} + C_{\rm sh} = 2(C_{\rm J} + C_{\rm sh}) + C + C_{\rm g}$, and inductance matrix:

$$[L^{-1}] = \frac{1}{2}\begin{bmatrix} 2/L^* + 1/L & -1/L & 0 & 0 & 0 & \dots & 0 \\ -1/L & 2/L & -1/L & 0 & 0 & \dots & 0 \\ 0 & -1/L & 2/L & -1/L & 0 & \dots & 0 \\ \vdots & \vdots & \ddots & \ddots & \ddots & \dots & 0 \\ 0 & 0 & 0 & -1/L & 2/L & -1/L & 0 \\ 0 & 0 & 0 & 0 & -1/L & 2/L & -1/L \\ 0 & 0 & 0 & 0 & 0 & -1/L & 1/L \end{bmatrix}.$$

Here $L^* = (\hbar/2e)^2/E_{\rm J}^*$ is an effective inductance associated with the weak link.

Let us define by $E_k = \hbar\omega_k$ the eigenvalues of $\hat{H}_{\rm t}$ and $\hat{a}_k^\dagger$ the corresponding creation operators associated to its normal modes. As $\hat{H}_{\rm t}$ is harmonic, one can write:

$$\hat{H}_{\rm t} = \sum_{k=0}^{N+1} \hbar\omega_k \hat{a}_k^\dagger \hat{a}_k, \tag{12}$$

$$\hat{\phi}_{\rm J} = \sum_{k=0}^{N+1} \phi_k (\hat{a}_k^\dagger + \hat{a}_k). \tag{13}$$

The renormalized Josephson energy $E_{\rm J}^*$ is obtained by minimizing the variational free energy:

$$\frac{d}{dE_{\rm J}^*}(F_{\rm t} + \langle \hat{H} - \hat{H}_{\rm t} \rangle_{\rm t}) = 0. \tag{14}$$

The first term is evaluated as follows:

$$\frac{dF_{\rm t}}{dE_{\rm J}^*} = -\frac{k_{\rm B}T}{Z_{\rm t}}\frac{dZ_{\rm t}}{dE_{\rm J}^*} = -\frac{k_{\rm B}T}{Z_{\rm t}}\sum_k \frac{d}{dE_{\rm J}^*}\left(e^{-E_k/k_{\rm B}T}\right) = \frac{1}{Z_{\rm t}}\sum_k \langle k|\,\frac{d\hat{H}_{\rm t}}{dE_{\rm J}^*}\,|k\rangle\, e^{-E_k/k_{\rm B}T} \tag{15}$$

$$= \frac{1}{Z_{\rm t}}\sum_k \langle k|\,\frac{\hat{\phi}_{\rm J}^2}{2}\,|k\rangle\, e^{-E_k/k_{\rm B}T} = \frac{\langle\hat{\phi}_{\rm J}^2\rangle_{\rm t}}{2} \tag{16}$$

where we used the fact that $\langle k|\,\hat{H}_{\rm t}\frac{d}{dE_{\rm J}^*}\,|k\rangle = 0$, which follows because $|k\rangle$ is a normalized eigenstate of $\hat{H}_{\rm t}$ and $\frac{d}{dE_{\rm J}^*}\,|k\rangle$ is orthogonal to $|k\rangle$. The second term in the variational free energy is

$$\frac{d}{dE_{\rm J}^*}\langle\hat{H}-\hat{H}_{\rm t}\rangle_{\rm t} = -\frac{E_{\rm J,bare}}{2}\frac{d}{dE_{\rm J}^*}\langle e^{i\hat{\phi}_{\rm J}}+e^{-i\hat{\phi}_{\rm J}}\rangle_{\rm t} - \frac{\langle\hat{\phi}_{\rm J}^2\rangle_{\rm t}}{2} - \frac{E_{\rm J}^*}{2}\frac{d}{dE_{\rm J}^*}\langle\hat{\phi}_{\rm J}^2\rangle_{\rm t}. \tag{17}$$

Inserting Eq. (16) and Eq. (17) in Eq. (14), one finds the following condition on $E_{\rm J}^*$:

$$E_{\rm J}^* = -E_{\rm J,bare}\frac{\frac{d}{dE_{\rm J}^*}(\langle e^{i\hat{\phi}_{\rm J}}+e^{-i\hat{\phi}_{\rm J}}\rangle_{\rm t})}{\frac{d}{dE_{\rm J}^*}\langle\hat{\phi}_{\rm J}^2\rangle_{\rm t}}. \tag{18}$$

Supplementary Note 5: Microscopic model

Let us now compute $\langle e^{i\hat{\phi}_{\rm J}}\rangle_{\rm t}$ using Eq. (13) and the Baker-Campbell-Hausdorff formula :

$$\langle e^{i\hat{\phi}_{\rm J}}\rangle_{\rm t} = \langle\exp\left(i\sum_{k=0}^M\phi_k(\hat{a}_k^\dagger+\hat{a}_k)\right)\rangle_{\rm t} = \langle\exp\left(i\sum_{k=0}^M\phi_k\hat{a}_k^\dagger\right)\exp\left(i\sum_{k=0}^M\phi_k\hat{a}_k\right)\rangle_{\rm t}\exp\left(-\frac{1}{2}\sum_{k=0}^M\phi_k^2\right) \tag{19}$$

$$= \langle\sum_{n\geq 0}\sum_{m\geq 0}\frac{i^n i^m}{n!m!}\left(\sum_{k=0}^M\phi_k\hat{a}_k\right)^n\left(\sum_{k=0}^M\phi_k\hat{a}_k\right)^m\rangle_t\exp\left(-\frac{1}{2}\sum_{k=0}^M\phi_k^2\right). \tag{20}$$

The terms where $n=m$ are the only one different from 0 :

$$\langle e^{i\hat{\phi}_{\rm J}}\rangle_{\rm t} = \sum_{m\geq 0}\frac{(-1)^n}{n!^2}\sum_{k_1...k_n}\phi_{k_1}...\phi_{k_n}\sum_{k_1'...k_n'}\phi_{k_1'}...\phi_{k_n'}\langle a_{k_1}^\dagger...a_{k_n}^\dagger a_{k_1'}...a_{k_n'}\rangle_t \tag{21}$$

$$= \sum_{n\geq 0}\frac{(-1)^n}{n!^2}n!\sum_{k_1...k_n}\phi_{k_1}^2...\phi_{k_n}^2\langle a_{k_1}^\dagger a_{k_1}\rangle_t...\langle a_{k_n}^\dagger a_{k_n}\rangle_t\exp\left(-\frac{1}{2}\sum_{k=0}^N\phi_k^2\right) \tag{22}$$

$$= \sum_{n\geq 0}\frac{1}{n!}\left(-\sum_{k=0}^N\left(n_k\phi_k^2\right)\right)^n\exp\left(-\frac{1}{2}\sum_{k=0}^N\phi_k^2\right) = \exp\left(-\sum_{k=0}^N(n_k+\frac{1}{2})\phi_k^2\right) = \exp\Big(-\langle\hat{\phi}_{\rm J}^2\rangle_{\rm t}/2\Big). \tag{23}$$

Wick's theorem has been used between Eq. (21) and Eq. (22), and $n_k = 1/[\exp(\hbar\omega_k/k_{\rm B}T)-1]$ is the Bose factor. One verifies easily that $\langle e^{-i\hat{\phi}_{\rm J}}\rangle_{\rm t} = \langle e^{i\hat{\phi}_{\rm J}}\rangle_{\rm t}$. We can finally simplify the term appearing in Eq. (18):

$$\frac{d}{dE_{\rm J}^*}\langle e^{i\hat{\phi}_{\rm J}}+e^{-i\hat{\phi}_{\rm J}}\rangle_{\rm t} = -e^{-\langle\hat{\phi}_{\rm J}^2\rangle_{\rm t}}\frac{d}{dE_{\rm J}^*}(\langle\hat{\phi}_J^2\rangle_{\rm t}) \tag{24}$$

so that $E_{\rm J}^*$ obeys the simple self-consistency relation:

$$\boxed{E_{\rm J}^* = E_{\rm J,bare}e^{-\langle\hat{\phi}_{\rm J}^2\rangle_{\rm t}/2} = E_{\rm J,bare}\exp\left[-\sum_{k=0}^N\phi_k^2(n_k+\tfrac{1}{2})\right]} \tag{25}$$

We finally present the procedure to compute the normal mode expansion coefficients ϕ_k, as obtained from the trial Hamiltonian $\hat{H}_{\rm t}$. The original charge and phase variables can be decomposed formally onto the normal modes:

$$\hat{n}_p = -\frac{i}{2\pi}\sqrt{\frac{R_Q}{2}}\sum_{k=1}^{N}[G]_{p,k}(\hat{a}_k - \hat{a}_k^\dagger), \tag{26}$$

$$\hat{\phi}_p = 2\pi\sqrt{\frac{1}{2R_Q}}\sum_{k=0}^{N}[R]_{p,k}(\hat{a}_k + \hat{a}_k^\dagger). \tag{27}$$

By imposing the canonical commutation relation for the bosonic operators and $[\hat{\phi}_p, \hat{n}_m] = i\delta_{pm}$, we obtain the following normalization condition on the matrices $[R]$ and $[G]$:

$$[G][R]^T = I. \tag{28}$$

Using Eq. (26) and Eq. (27) in $\hat{H}_t$, we obtain :

$$\hat{H}_t = \frac{\hbar}{4}\sum_{m,p=1}^{N+1}(\hat{a}_p^\dagger + \hat{a}_p)[G^T C^{-1} G]_{p,m}(\hat{a}_m^\dagger + \hat{a}_m) - (\hat{a}_p^\dagger - \hat{a}_p)[R^T L^{-1} R]_{p,m}(\hat{a}_m^\dagger - \hat{a}_m). \tag{29}$$

In order to recover the usual harmonic form (12) of $\hat{H}_t$, we firstly impose:

$$[L^{-1}C^{-1}G] = [G\Omega^2], \tag{30}$$

implying that the columns of $[G]$ contain the right-eigenvectors of $[L^{-1}C^{-1}]$, $[\Omega]$ being the positive definite diagonal matrix such that $[\Omega^2]$ contains the eigenvalues of $[L^{-1}C^{-1}]$. Then we note that

$$\begin{aligned}[G^T C^{-1}][L^{-1}C^{-1}] &= [G^T C^{-1} L^{-1}][C^{-1}] \\ &= [\Omega^2 G^T][C^{-1}] = [\Omega^2][G^T C^{-1}]\end{aligned} \tag{31}$$

i.e. the rows of $[G^T C^{-1}]$ contain the left-eigenvectors of $[L^{-1}C^{-1}]$, implying that we can take $[G^T C^{-1} G]$ as diagonal. We have not yet specified the normalization of the columns of G. We do so now by imposing

$$[G^T C^{-1} G] = [\Omega] \tag{32}$$

From Eq. 28 then follows that $[R^T] = [\Omega^{-1} G^T C^{-1}]$. Using this together with Eq. (30), we then derive that also

$$[R^T L^{-1} R] = [\Omega] \tag{33}$$

Substitution into Eq. (29) then yields

$$H_{\rm t} = \hbar\sum_{p=1}^{N+1}\omega_p(a_p^\dagger a_p + 1/2), \tag{34}$$

with $\omega_p = [\Omega]_{pp}$. Once the $[L^{-1}C^{-1}]$ eigenvalue problem has been numerically solved, we can express the phase across the weak link in terms of the normal mode amplitudes

$$\phi_k = \pi\sqrt{\frac{2}{R_{\rm Q}}}[R]_{0,k}, \tag{35}$$

so that the final self-consistent equation for $E_{\rm J}^*$ is :

$$E_{\rm J}^* = E_{\rm J}\exp\left(-2\pi^2\sum_{k=0}^{N}\frac{[R]_{0,k}^2}{R_{\rm Q}}\frac{1+2n_k}{2}\right). \tag{36}$$

In practice, we determine $E_{\rm J}^*$ from the Hamiltonian formalism described here. Once the value has been determined (which in general depends also on temperature), it can be inserted in a full ABCD calculation [3], since the effect of the capacitive coupling to the output ports is very small in practice.

Supplementary Note 6: Phase shift induced by the small Josephson junction

Now that we have obtained the best harmonic approximation of $\hat{H}$ by solving (36) self-consistently, we can investigate the effect of the small junction on the odd modes with respect to decoupled even modes. In frequency domain, the equations of motion for the classical phases $\phi_{j,-}$ are given by :

$$[L^{-1}][\phi_-] = [C][\phi_-][\Omega^2], \tag{37}$$

with $[L^{-1}]$ and $[C]$ the inductance and capacitance matrices for the odd modes, and the columns of the matrix $[\phi]$ tabulate the phase configuration for different frequencies. The even modes form stationary cosine waves along the chain:

$$[\phi_+]_{l,k} = N\cos[k(l+1/2)], \tag{38}$$

with $l = 0, 1, 2...$ the position in the chain and k the wavenumber. The dispersion relation reads

$$k = 2\,\mathrm{arccot}\sqrt{\left(\frac{4C}{C_\mathrm{g}}+1\right)\left(\left[\frac{\omega_\mathrm{p}}{\omega(k)}\right]^2-1\right)}, \tag{39}$$

with $\omega_\mathrm{p} = 1/\sqrt{L}(C + C_\mathrm{g}/4)$ the plasma frequency of the chain.

In presence of the small junction (treated at the SCHA level), the odd modes have the same dispersion relation but experience an additional phase shift θ (we omit in our notation the fact that $\theta = \theta_k$ depends implicitely on k):

$$[\phi_-]_{l,k} = N\cos[k(l+1/2)-\theta]. \tag{40}$$

The phase shift is determined from equation of motion that links sites 0 and 1

$$\left(\frac{2}{L^*}+\frac{1}{L}\right)[\phi_-]_{0,k} - \frac{1}{L}[\phi_-]_{1,k} = \omega^2(C_\Sigma[\phi_-]_{0,k} - C[\phi_-]_{1,k}), \tag{41}$$

$$\tag{42}$$

which we can rewrite using Eq. (40) as

$$\cos(k/2-\theta) = \lambda\cos(3k/2-\theta) \tag{43}$$

where

$$\lambda = \frac{1-\omega^2 CL}{(1+\frac{2L}{L_J^*}) - \omega^2 C_\Sigma L}. \tag{44}$$

In the case where the junction is saturated (either at strong driving power, or for large thermal fluctuations), we have $E_J^* = 0$, and we use:

$$\lambda = \frac{1-\omega^2 CL}{1-\omega^2 C_\Sigma L}. \tag{45}$$

Solving for θ, we find

$$\theta = k + \arctan\left[\frac{(1-\lambda)\mathrm{X}}{1+\lambda}\right] \tag{46}$$

where

$$X = \cot\left(\frac{k}{2}\right) = \sqrt{\left(\frac{4C}{C_\mathrm{g}}+1\right)\left[\left(\frac{\omega_\mathrm{p}}{\omega}\right)^2-1\right]}. \tag{47}$$

Supplementary Note 7: Splitting between odd and even modes

Now that we have the analytic expression (46) for the phase shift induced by the small non-linear junction, we will see how it translates into the splitting between odd and even modes. For simplicity, we will assume here that C_c and $C_{c,I}$ are big enough so that we can consider the last site N as grounded:

$$k_n(N+1/2) - \theta_n = \pi(n - \frac{1}{2}), \tag{48}$$

with θ_n the phase shift for the mode n, so that :

$$k_n = k_n^\circ + \frac{\theta_n}{N+1/2}, \tag{49}$$

with k_n° the wave vector of the mode n in the bare chain (corresponding to the uncoupled even modes in the experiment). Using the dispersion relation, we find at order $1/N$:

$$\omega(k_n) = \omega\left(k_n^\circ + \frac{\theta_n}{N+1/2}\right) = \omega(k_n^\circ) + \frac{\theta_n}{N}\frac{\partial\omega(k)}{\partial k}\Big|_{k=k_n^\circ} + O(N^{-2}). \tag{50}$$

We also have for the bare modes:

$$\omega(k_{n+1}^\circ) = \omega(k_n^\circ) + \frac{\pi}{N}\frac{\partial\omega(k)}{\partial k}\Big|_{k=k_n^\circ} + O(N^{-2}) \tag{51}$$

Using Eq. (50) and Eq. (51), we obtain the connection between the relative odd-even splitting S induced by the small junction on the odd modes and the associated phase shift θ_n on mode n:

$$\theta_n = \pi\frac{\omega(k_n) - \omega(k_n^\circ)}{\omega(k_{n+1}^\circ) - \omega(k_n^\circ)} = \pi S. \tag{52}$$

To make sure that approximating the site N as grounded is valid, we computed numerically the exact splitting obtained with and without these pads, using a full ABCD matrix calculation (shown in Supplementary Figure 4 with the parameters of sample B), and found very little effect of this approximation. In addition, we find that the theoretical phase shift Eq. (46), valid for an infinite chain and shown by the black solid line in Supplementary Figure 4 compares quantitatively to the ABCD simulations (dots) of the real device.

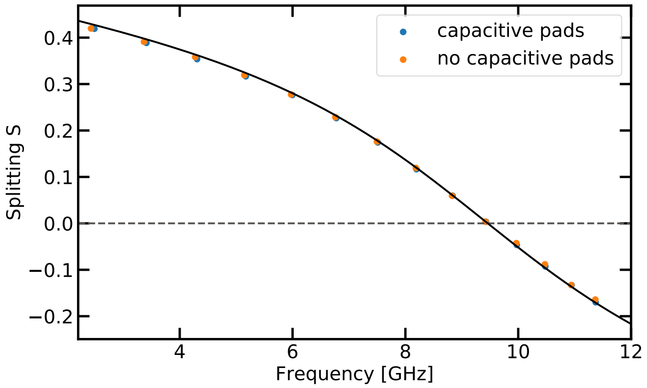

375.pdf

Supplementary Figure 4. **Comparision between the analytical phase shift and the simulated even-odd splitting.** The normalized phase shift θ_n/π from formula (46) is in excellent agreement with full ABCD simulations of sample B (dots), confirming also a very small effect of the coupling pads to the output ports.

In the infinite system, the phase shift θ becomes a continuous function of frequency ω. It vanishes at the renormalized frequency

$$\omega_J^* = \frac{1}{\sqrt{L^*(C_\mathrm{J} + C_\mathrm{sh})}} \tag{53}$$

of the weak link, as can be seen as follows. When $\theta = 0$, Eq. (46) can be rewritten as

$$\cot k = \frac{\lambda + 1}{(\lambda - 1)X}. \tag{54}$$

From the definition of X follows that $\cot k = (X^2 - 1)/2X$, and furthermore, that

$$\frac{X^2 - 1}{2} = 2\frac{1 - LC\omega^2}{LC_\mathrm{g}\omega^2} - 1. \tag{55}$$

Using the definition (44) of λ and that of C_Σ, we reduce Eq. (54) to

$$\frac{1 - LC\omega^2}{LC_\mathrm{g}\omega^2} = \frac{1 - LC\omega^2}{\omega^2 L[2(C_\mathrm{J} + C_\mathrm{sh}) + C_\mathrm{g}] - 2L/L^*} \tag{56}$$

implying that

$$\omega^2(C_J + C_\mathrm{sh}) - 1/L^* = 0 \tag{57}$$

and hence $\omega = 1/\sqrt{L^*(C_\mathrm{J} + C_\mathrm{sh})} \equiv \omega_\mathrm{J}^*$.

Supplementary Note 8: Fitting the experimental splittings

We present in Supplementary Figure 5 the frequency-dependent splittings extracted from the analysis of the even-odd mode pairs (see Supplementary Figure 3), shown as dots for our three samples and various temperatures. Each of this data set is then fitted to the analytical formula (46), $L^*(T)$, or equivalently $E_\mathrm{J}^*(T)$ being the fitting parameter. The range of investigated temperature is restricted below 130 mK, since at too high temperatures, thermal fluctuations are so strong that the SCHA treatment breaks down. We find in Supplementary Figure 5 that the lineshape of the splitting is well reproduced by our calculations. The location of the zero of the splitting also allows to extract the value of the renormalized frequency ω_J^* of the small junction, a key quantity that is discussed in detail in the main text.

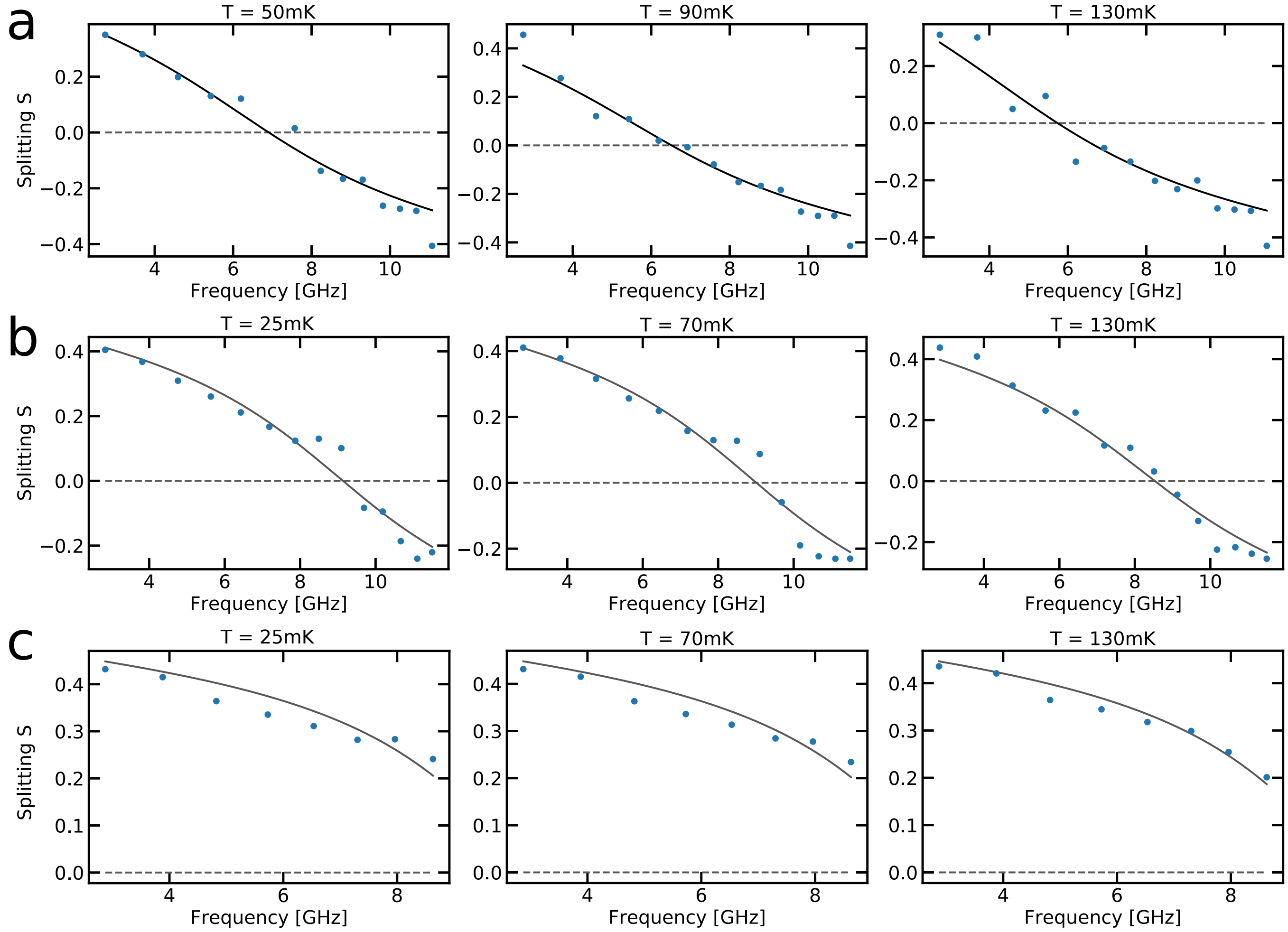

Supplementary Figure 5. **Analysis of the experimental even-odd splitting.** The extracted experimental splitting are shown as dots for our three samples (**a** is for sample A, **b** is for sample 375 and **c** is for sample 450) and various temperatures as indicated. Formula (46) is fitted (black solid lines), allowing the extraction of the renormalized frequency ω_J^*.

Supplementary Note 9: Estimation of the shunting capacitance

To determine a value of the unknown shunting capacitance $C_{\rm sh}$, we devised an original saturation technique. At high enough power, the fluctuations across the small junction can be so large that $E_{\rm J}^*$ renormalizes to zero, decoupling effectively the dynamics of the two chains, except for the remaining effect of $C_{\rm sh}$ and $C_{\rm J}$. We can thus use formula (45), and since $C_{\rm J}$ is known by design, one can directly infer $C_{\rm sh}$ from an analysis of the even-odd splitting at high power. The evolution of the transmission as a function of power, and the resulting splittings are shown in Supplementary Figure 6. From that measurement one can infer that $C_{\rm sh}$ slightly increases (see Table I in the main text) when the size of the junction is increased, which is the expected behavior.

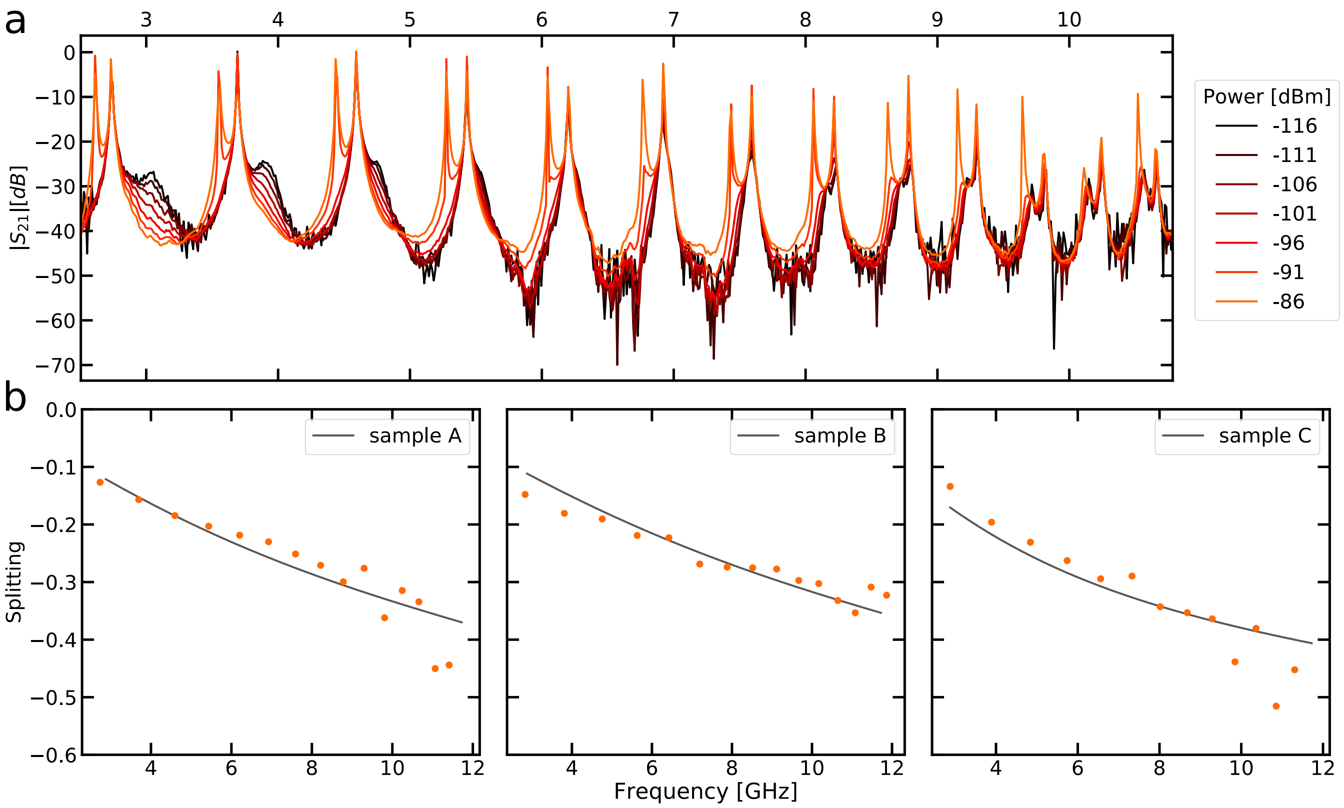

Supplementary Figure 6. **Power scan.** Panel **a** shows the transmission $|S_{21}|$ of sample 300 as a function of the frequency for different power values imposed to the sample. Panel **b** shows the extracted splitting for our three samples, fitted from Eq. (45), allowing to extract the shunting capacitance $C_{\rm sh}$.

Supplementary Note 10: Extracting the parameters of the chain

In this section we discuss how the parameters of the chain are extracted. The chains used in our samples are made out of SQUIDs. Consequently, the inductance of the chains are given by :

$$L = \frac{L_{\rm J_{ch,min}}}{\sqrt{\cos^2(\Phi_{\rm C}/\Phi_0) + d^2 \sin^2(\Phi_{\rm C}/\Phi_0)}} \tag{58}$$

with $\Phi_{\rm C}$ the flux in the SQUID loops and d the asymmetry of the SQUID junctions [2]. As we can neglect the effect output port capacitances, the dispersion relation of the even modes is given by (39), which can be expressed as a function of ω:

$$\omega(k) = \frac{1}{\sqrt{L(\Phi_{\rm C})C}} \sqrt{\frac{1-\cos(ka)}{1-\cos(ka) + \frac{C_{\rm g}}{2C}}} \tag{59}$$

From Eq. (58), the free spectral range (namely the energy difference between two consecutive modes) is decreasing when $\Phi_{\rm C}/\Phi_0$ goes to $\pi/2$. This behavior is clearly seen in Supplementary Figure 7. One can also notice the absence

of artifacts around $\Phi_C = 0$, which means that the chain is homogeneous and relatively exempt of disorder. By doing a two-tone spectroscopy at $\Phi_C = 0$, we can measure precisely the dispersion of the even modes up to 14GHz. From Eq. (59), we find C_g and L for the three sample, C being known by design. This method allows an in-situ determination of the chain parameters.

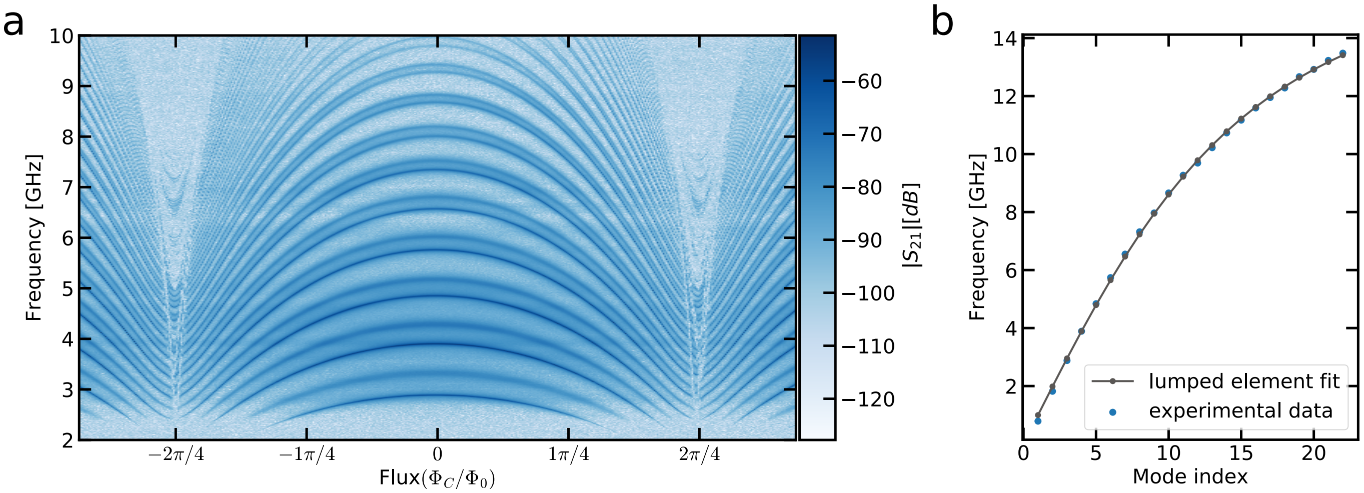

Supplementary Figure 7. **Determination of the chain parameters.** Panel **a** shows the transmission $|S_{21}|$ of sample C as a function of the frequency for different flux in the SQUIDs of the chain. Panel **b** shows the dispersion relation for the even modes extracted at half flux quantum.

Supplementary Note 11: Perturbative treatment of the non linearity

The perturbative treatment is commonly used in circuit-QED whenever one needs to consider the non-linearity induced by a Josephson junction in a superconducting circuit [4, 5]. As a first step, the tunnelling energy $E_{\mathrm{J,bare}}(1-\cos\hat{\phi}_{\mathrm{J}})$ is approximated by its harmonic approximation $E_{\mathrm{J,bare}}\hat{\phi}_{\mathrm{J}}^2/2$, leading to an effective quadratic Hamiltonian (without any renormalization) $\hat{H}^{\mathrm{lin}} = \sum_{k=0}^{M} \hbar\omega_k^{\mathrm{lin}}\hat{a}_k^\dagger\hat{a}_k$ and a mode decomposition of the phase fluctuating across the weak link $\hat{\phi}_{\mathrm{J}} = \sum_{k=0}^{M}\phi_k^{\mathrm{lin}}(\hat{a}_k^\dagger + \hat{a}_k)$. The non linearity is then reintroduced at quartic level:

$$\hat{H} \simeq \hat{H}^{\mathrm{lin}} - \frac{E_{\mathrm{J}}}{24}\hat{\phi}_{\mathrm{J}}^4. \tag{60}$$

This quartic perturbation renormalizes the modes at order E_{J}:

$$\omega_k^* = \omega_k - [K]_{k,k} + \sum_{j\neq k}[K]_{\mathrm{k,j}}, \tag{61}$$

$$K_{k,j} = E_{\mathrm{J}}\left(\frac{\pi}{R_{\mathrm{Q}}}[R]_{0,k}[R]_{0,j}\right)^2. \tag{62}$$

$[K]$ is known as the Kerr matrix [6]. Using this formalism, one can compute the splitting between odd and even modes (see Supplementary Figure 8). Fitting with the phase shift formula (46), we deduce the renormalized Josephson energy from the Kerr theory $E^*_{\mathrm{J,Kerr}}$, which can be compared to the SCHA estimate $E^*_{\mathrm{J,SCHA}}$ and the bare value $E_{\mathrm{J,bare}}$. For sample B, we find $E_{\mathrm{J,bare}} = 5.61$ GHz, $E_{\mathrm{J,Kerr}} = 4.47$ GHz and $E_{\mathrm{J,SCHA}} = 3.30$ GHz. The renormalization of $E_{J,\mathrm{bare}}$ from the SCHA acquires a clear non-perturbative character, which the standard Kerr approach is unable to predict quantitatively. This confirms that our device operates in the many-body regime, and cannot be described by standard approaches such as black-box-quantization [4].

]

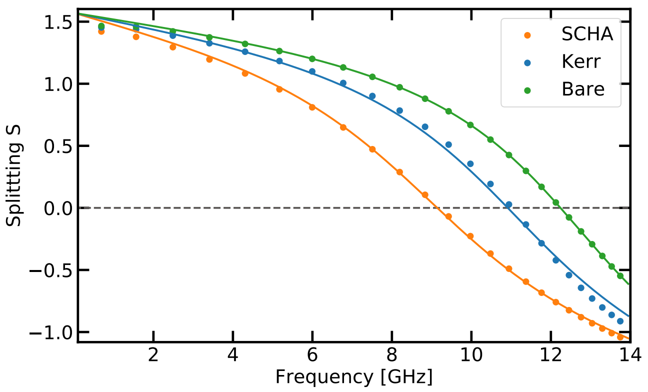

Supplementary Figure 8. **Comparing various quantum approaches of many-body circuits.** The even-odd splittings for parameters of sample B are obtained from three numerical approaches (dots): a bare formalism using the fully linearized Josephson Hamiltonian (green), a Kerr approach incorporating the quartic correction (blue), and the self-consistent harmonic approximation taking into account the full cosine form of the potential (orange). Solid lines are the fits from the phase shift formula (46) allowing to extract the resonance frequency of the junction, and its associated Josephson energy.

[1] P. Joyez, Physical Review Letters **110**, 312 (2013).
[2] J. Puertas Martinez, S. Leger, N. Gheeraert, R. Dassonneville, L. Planat, F. Foroughi, Y. Krupko, O. Buisson, C. Naud, W. Hasch-Guichard, S. Florens, I. Snyman, and N. Roch, npj Quantum Information , 1 (2019).
[3] D. M. Pozar, *Microwave engineering* (John Wiley & Sons, 2009).
[4] S. E. Nigg, H. Paik, B. Vlastakis, G. Kirchmair, S. Shankar, L. Frunzio, M. H. Devoret, R. J. Schoelkopf, and S. M. Girvin, Physical Review Letters **108**, 260 (2012).
[5] T. Weissl, B. Küng, E. Dumur, A. K. Feofanov, I. Matei, C. Naud, O. Buisson, F. W. J. Hekking, and W. Guichard, Physical Review B **92**, 104508 (2015).
[6] Y. Krupko, V. D. Nguyen, T. Weissl, E. Dumur, J. Puertas, R. Dassonneville, C. Naud, F. W. J. Hekking, D. M. Basko, O. Buisson, N. Roch, and W. Hasch-Guichard, Phys Rev B **98**, 094516 (2018).
